# Breastfeeding in patients with Gaucher disease: Is taliglucerase alfa safe?

**DOI:** 10.1016/j.ymgmr.2018.11.004

**Published:** 2019-01-16

**Authors:** Livia Paskulin, Alícia Dorneles Dornelles, Amanda Quevedo, Tatiele Nalin, Kristiane Michellin Tirelli, Filippo Vairo, Ida Vanessa Doederlein Schwartz

**Affiliations:** a Medical Genetics Service, Hospital de Clínicas de Porto Alegre, Porto Alegre, Brazil; b Post-Graduation Program in Genetics and Molecular Biology, Universidade Federal do Rio Grande do Sul, Porto Alegre, Brazil; c Post Graduation Program in Clinical Medicine, Universidade Federal do Rio Grande do Sul, Porto Alegre, Brazil; d Center for Individualized Medicine, Mayo Clinic, Rochester, MN, USA; e Department of Clinical Genomics, Mayo Clinic, Rochester, MN, USA; f Genetics Department, Universidade Federal do Rio Grande do Sul, Porto Alegre, Brazil

Dear Editor

In 2014 [1], we shared our experience on breastfeeding and enzyme replacement therapy (ERT) with imiglucerase in Gaucher disease (GD). Our results were in accordance with *Sekijima* et al. [2], and showed that activity of β–glucocerebrosidase in human breast milk from a patient with type 1 GD on ERT with imiglucerase (patient 1) is much lower than the levels of natural β–glucocerebrosidase in breast milk from a healthy woman. There is no report in the literature regarding the use of taliglucerase alfa (a recombinant β–glucocerebrosidase produced in carrots cells) in breastfeeding patients with GD. Herein, we report on our experience with this recombinant enzyme during breastfeeding.

A 20-year-old female (patient 2) was diagnosed with GD after a liver biopsy performed due to hepatosplenomegaly (*GBA1* genotype: N370S/L444P). Taliglucerase alfa 15 U/kg biweekly was initiated, following the Brazilian Ministry of Health recommendations [3]. She was diagnosed as being 6-week pregnant after the seventh infusion. Since there were no recommendations regarding the treatment with taliglucerase alfa during pregnancy, ERT was maintained. She was referred for prenatal care in a specialized hospital, where she followed all recommendations regarding diet and vitamin supplementations, and did not experience any complication during pregnancy. She gave birth through vaginal delivery to a full-term healthy baby boy at 39 weeks of pregnancy, and exclusively on-demand breastfeeding was initiated and maintained through 10 months. Patient received calcium supplementation (500 mg/day) and maintained her regular infusions with a dosage of 20 U/kg every other week.

One month after delivery, blood samples were collected before and 30 min after her scheduled infusion of taliglucerase alfa, and we did the same analysis in blood and breast milk we have done for patient 1: breast milk samples were collected before, immediately after and 30 min after the infusion. β–glucocerebrosidase activity was measured in leukocytes and in breast milk as described by *Peters* et al. [4] (Table 1).

**Table 1:** β–glucocerebrosidase activity in leukocytes and breast milk from a patient with Gaucher disease before and after infusion of imiglucerase 30 U/kg (patient 1) and taliglucerase alfa 20 U/kg (patient 2).

Variable	β-glucocerebrosidase activity	
	Patient 1 (Dornelles et al. [1])	Patient 2 (Current Study)
Leukocytes (nmol/h/mg prot)^a^		
Before infusion	0.62	1.3
After infusion^b^	7.5	3.5
Breast milk (nmol/h/mL)^c^		
Before infusion	2	3.9
After infusion	3	7.1
30 min after infusion	4	7.2

a Normal Range Value = 10–45 nmol/h/mg prot. Samples were collected immediately after infusion, which has an approximate duration of 2 h.

b In patient 1, collected right after infusion; in patient 2, collected 30 min after infusion.

c β–glucocerebrosidase activity measured in a control milk sample was 42 nmol/h/mL.

According to our data, activity of β–glucocerebrosidase in human breast milk from patient 2 is also much lower than the levels of natural β–glucocerebrosidase in breast milk from a healthy woman, suggesting that the levels of recombinant enzymes excreted in human breast milk is very low. For this reason, we believe that taliglucerase alfa should not be contraindicated to women with GD during breastfeeding. Actually, we advocate that ERT should be indicated to these women, together with calcium supplementation, since it is a period in which the need for calcium and the bone remodeling increases [5].

## Conflicts of interest

The authors indicate that they have no conflicts of interest regarding the content of this article.
